# Optimal LC-MS metabolomic profiling reveals emergent changes to monocyte metabolism in response to lipopolysaccharide

**DOI:** 10.3389/fimmu.2023.1116760

**Published:** 2023-03-23

**Authors:** Emma Leacy, Isabella Batten, Laetitia Sanelli, Matthew McElheron, Gareth Brady, Mark A. Little, Hania Khouri

**Affiliations:** ^1^ Trinity Translational Medicine Institute, Faculty of Health Sciences, Trinity College Dublin, Dublin, Ireland; ^2^ Faculty of Health Medicine and Life Sciences, Maastricht University, Maastricht, Netherlands; ^3^ Trinity Health Kidney Centre, Tallaght University Hospital, Dublin, Ireland; ^4^ Agilent Technologies, Stockpoty, England, United Kingdom

**Keywords:** monocyte, metabolomics, LPS, LC-MS, data normalization

## Abstract

**Introduction:**

Immunometabolism examines the links between immune cell function and metabolism. Dysregulation of immune cell metabolism is now an established feature of innate immune cell activation. Advances in liquid chromatography mass spectrometry (LC-MS) technologies have allowed discovery of unique insights into cellular metabolomics. Here we have studied and compared different sample preparation techniques and data normalisation methods described in the literature when applied to metabolomic profiling of human monocytes.

**Methods:**

Primary monocytes stimulated with lipopolysaccharide (LPS) for four hours was used as a study model. Monocytes (n=24) were freshly isolated from whole blood and stimulated for four hours with lipopolysaccharide (LPS). A methanol-based extraction protocol was developed and metabolomic profiling carried out using a Hydrophilic Interaction Liquid Chromatography (HILIC) LC-MS method. Data analysis pipelines used both targeted and untargeted approaches, and over 40 different data normalisation techniques to account for technical and biological variation were examined. Cytokine levels in supernatants were measured by ELISA.

**Results:**

This method provided broad coverage of the monocyte metabolome. The most efficient and consistent normalisation method was measurement of residual protein in the metabolite fraction, which was further validated and optimised using a commercial kit. Alterations to the monocyte metabolome in response to LPS can be detected as early as four hours post stimulation. Broad and profound changes in monocyte metabolism were seen, in line with increased cytokine production. Elevated levels of amino acids and Krebs cycle metabolites were noted and decreases in aspartate and β-alanine are also reported for the first time. In the untargeted analysis, 154 metabolite entities were significantly altered compared to unstimulated cells. Pathway analysis revealed the most prominent changes occurred to (phospho-) inositol metabolism, glycolysis, and the pentose phosphate pathway.

**Discussion:**

These data report the emergent changes to monocyte metabolism in response to LPS, in line with reports from later time points. A number of these metabolites are reported to alter inflammatory gene expression, which may facilitate the increases in cytokine production. Further validation is needed to confirm the link between metabolic activation and upregulation of inflammatory responses.

## Introduction

### Immunometabolism

Immunometabolism examines the links between immune cell function and metabolism. The field has burgeoned from early discoveries linking succinate accumulation to interleukin-1β (IL-1β) production in macrophages (1, 2), and we now know that metabolic pathways and immunologic functions are a complex concatenation of processes with distinct, context-dependent outputs. There are two primary pathways used by cells to generate energy in the form of adenosine triphosphate (ATP): the glycolytic pathway (glycolysis) and oxidative phosphorylation (OXPHOS) (3). Glycolytic intermediates can be diverted to feed multiple metabolic pathways including the pentose phosphate pathway (PPP), tricarboxylic acid (TCA) cycle, and lipid synthesis pathways (3). Although glycolysis is less energy efficient than OXPHOS in terms of ATP production, it is often adopted by immune cells as a rapid means of generating energy during acute activation, in a process called the “Warburg effect” (4).

### Monocytes and their role in inflammation

Monocytes are myeloid cells accounting for approximately 10% of circulating leukocytes, whose primary role is immune defence. These cells are derived from myeloid bone marrow precursors and can transmigrate into tissues to differentiate into macrophages or dendritic cells. However, these cells are more than just a precursor, and are becoming more recognised for their heterogeneous responses to inflammation and roles in tissue repair and trained immunity (5). Prior to differentiation, monocytes are typically pro-inflammatory cells. They can rapidly propagate inflammation directly by phagocytosis and antigen presentation, as well as through release of cytokines, reactive oxygen species (ROS) and other inflammatory signalling molecules (6, 7). During inflammation (sterile or infectious), circulating monocytes extravasate to inflamed tissues *via* the leukocyte recruitment cascade, and can rapidly become the dominant infiltrating mononuclear phagocyte in damaged tissues and draining lymph nodes.

### Monocyte metabolism

To perform these diverse functions, monocytes need dynamic energy processing systems. Monocytes exist in a “metabolically poised” state (8, 9), where they can react rapidly and specifically to different stimuli. These cells are also metabolically flexible and can utilise diverse fuel sources to fuel inflammation (10). Even in conditions of low oxygen – such as arthritic joints – these cells have been shown to adapt their metabolism to continue to preserve inflammatory cytokine production (11, 12). Glucose does appear to be a requirement for monocyte activation (13), differentiation (14), and cytokine production (15, 16). However, the way glucose is utilised during its breakdown may be of greater importance for cellular function. Glycolysis could be creating substrates for DNA (via the PPP) and cell membrane (via lipid metabolism) synthesis to facilitate cell growth and differentiation (17). Alternatively, glucose breakdown could be directed towards the TCA cycle, where fumarate and alpha-ketoglutarate can alter DNA methylation and gene expression (18).

Monocytes have been most closely studied in the context of LPS activation. LPS-stimulated TLR4 initiates a signalling cascade *via* MyD88 or TRIF leading to NF-κB/MAPK/IRF5 or IRF3 activation, respectively (19). Depleting NAD+ in monocytes inhibits TLR4 signalling, ultimately abrogating IL-1β production and highlighting a dependence on metabolic machinery (20). Lachmandas et al. investigated the differential metabolomic effects of various microbial stimulants in human monocytes and found diverse metabolic and inflammatory phenotypes (15). LPS did increase glycolysis rates at the expense of OXPHOS, however Pam3CysK4 and other bacterial lysates favoured increased oxygen consumption (15). *Candida* albicans (*C*. albicans) stimulation for instance led to sustained increases in both glycolysis and OXPHOS (16). A study replicating the effects of sepsis in monocyte-like THP1 cells detailed the switch from anabolic energy consumption during early immune activation (0-8h), to a catabolic energy-conserving process during immune deactivation (24-48h), before re-establishing energy homeostasis during resolution (48-96h) (21).

## Aims

Investigations into primary monocyte metabolism have thus far focused on metabolomic profiling after 24 hours of stimulation or more (11, 12, 15, 16, 22). In addition, none of these investigations have provided sufficient detail of how to replicate their sample processing of LC-MS methods. In this work, we set out to optimise and standardise LC-MS protocols for primary human monocytes and to define the early metabolomic changes that occur with LPS stimulation.

## Methods

### Blood collection and PBMC preparation

Fresh blood samples were taken from patients attending the outpatient’s haemochromatosis clinic in St. James’ Hospital. Whole blood donations were collected in CPDA-1 anti-coagulant bags (Fannin Scientific, MSE6500L) from consenting donors with no known infections and who disclosed their age and sex. PBMCs were isolated by density gradient centrifugation. Briefly, blood was added to 2% dextran (in PBS) mixed thoroughly at a 1:3 ratio and incubated for 30 mins at room temperature. This step was necessary to decrease the total blood volume while still ensuring sufficient monocyte numbers for optimisation studies, and can be omitted from future studies with a lower starting blood volume. The supernatant layer was removed and layered onto Lymphoprep™ and centrifuged at 400g for 25 mins with minimal acceleration and no breaking. The PBMC layer was aspirated and cells washed twice before resuspension in MACS buffer (PBS + 2mM EDTA + 0.5% BSA).

### Monocyte isolation and stimulation

Monocytes were isolated from PBMCs by CD14+ magnetic bead isolation. 100μL of anti-CD14 microbeads (Miltenyi Biotec,130-050-201) were added to 2.9mL PBMCs in MACS buffer and cells were incubated for 15 minutes at 4°C. CD14+ cells were eluted using an LS column and counted. The concentration was adjusted to 1x10^6^/mL in complete RPMI (RPMI + 10% FCS + 100U/mL streptomycin + 1mg/mL penicillin). Monocytes were seeded at 1x10^6^ cells/mL in a 24-well plate and left to rest at 37°C with 5% CO_2_ for at least 30 mins before stimulation. Cells were stimulated with 5μg/mL of monoclonal anti-MPO (Meridian BioSciences, H87207M) or anti-PR3 (Merck, MABT340) for 4 hours at 37°C with 5% CO_2_. In some experiments, monocytes were pre-treated with CBR-5884 (Sigma, SML1656) for 30 minutes before LPS stimulation. At the end of the stimulation, plates were centrifuged at 400g for 7 mins and supernatants removed for cytokine or cytotoxicity assay (Lactate dehydrogenase assay, LDH). 5x10^6^ monocytes were processed for metabolomic analysis.

### Measurement of cytokines and cytotoxicity assay

DuoSet ELISA kits (R&D Systems) were used to measure cytokine production in supernatants from ANCA-stimulated monocytes. Supernatants were diluted 1:5, 1:25, and 1:50 for tumour necrosis factor (TNF)-α (DY210), IL-1β (DY201), and IL-6 (DY206) assays, respectively. Quantification of LDH in post-stimulation cell supernatants was performed using the CytoTox 96^®^ Non-Radioactive Cytotoxicity Assay (Promega, G1780) to assess cell death/viability. Both protocols were performed as per manufacturer’s instructions.

### Sample preparation for LC-MS

**Figure 1:**
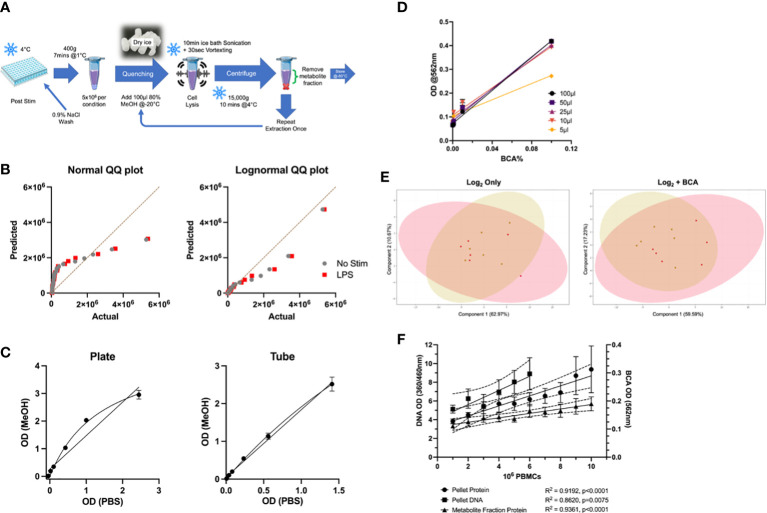
Optimisation of LC-MS Protocols for Metabolomic Profiling of Primary Monocytes. **(A)** Overview of optimised sample preparation protocol. **(B)** QQ plots of targeted metabolomics data (n=24) for normality testing by Anderson-Darling test, D’agostino & Pearson test, Shapiro-Wilk test, and Kolmogorov-Smirnov test. **(C)** Comparison of plate and tube protocols for BCA assay in various protein levels in PBS and MeOH. **(D)** Comparison of metabolite fraction (80% MeOH) volume used for protein determination by BCA assay. **(E)** PCA plots comparing log2 transformed to BCA-normalised targeted metabolomics data in unstimulated (gold) and LPS-stimulated (red) monocytes. **(F)** Comparison of biomass normalisation methods in PBMCs for metabolomic data normalisation. Protein levels of different cell numbers in the metabolite and pellet fractions were quantified by BCA assay, and DNA levels in the cell pellet were also measured. LC-MS, Liquid chromatography mass spectrometry; BCA, bicinchoninic acid; MeOH, methanol; PCA, Principal Component Analysis.

An optimised sample preparation protocol was used to extract metabolites from isolated monocytes post-stimulation (see **Figure 1A**). Following cell stimulation, 5x10^6^ treated monocytes were washed with ice-cold 0.9% NaCl and centrifuged in 1.5mL Eppendorf tubes at 400g for 7 mins. Supernatants were removed completely, and samples were quenched on dry ice. 100μL of ice cold 80% ACS reagent-grade methanol, (MeOH, Acros Organics, 10607221) was added, and cells were vortexed for 30 secs, then sonicated in an ice bath for 1 min, and vortexed for another 30secs. Samples were centrifuged at 16,000g for 10 mins at 4°C and the metabolite fraction (supernatant) removed for LC-MS analysis. The extraction procedure was repeated twice for a final metabolite fraction volume of 200μL. 5μL of metabolite fraction for each sample was added to an Eppendorf tube immediately after extraction to generate pooled quality control (PQC) samples. Extraction blanks were prepared using empty Eppendorf tubes as a negative control. Samples were stored at -80°C until analysis. A 50µM synthetic standard mix containing amino acids (Sigma, A9906), TCA cycle intermediates (Sigma, ML0010), and other metabolites of interest was formulated in 80% MeOH (see **Table S1**). To limit variations in metabolite abundances, all samples were processed on the same day with the same batch of reagents.

### Modified BCA assay for measurement of residual protein in metabolite fraction

Protein levels in metabolite fraction (and cell pellets) were measured using the Pierce™ BCA Protein Assay (Thermo Scientific, 23227). A top standard of 200µg/mL (300nM) bovine serum albumin (BSA) in was prepared in 80% MeOH (Acros Organics, 10607221) and serially diluted 1:2 down to 1.56µg/mL plus a negative blank sample. BCA Working Reagent was prepared by diluting Reagent B 1:50 with BCA Reagent A. 100μL of BCA Working Reagent was added to each well and the plate was mixed briefly on a plate shaker. The plate was sealed tightly with parafilm and left to incubate at 37°C for 18 hours. After cooling to room temperature, the absorbance was measured at 562nm with an Epoch™ Microplate Spectrophotometer (BioTek Instruments, Inc.).

### LC-MS metabolomic analysis

LC-MS metabolomics was performed as described by Hsiao et al. (23) with minor modifications. Samples were analysed on an Agilent 6546 Q-TOF (G6546A) paired with an Agilent 1290 Infinity LC. A 150mm×2.1mm InfinityLab Poroshell 120 HILIC-Z column (Agilent Technologies, 683775-924) was used and column temperature was maintained at 25°C. Mobile phase A consisted of 10mM ammonium acetate, pH 9.0 in water plus 5µM InfinityLab Deactivator Additive (Agilent Technologies, 5191-4506); and mobile phase B was 10mM ammonium acetate, pH 9.0 in 90% acetonitrile. The instrument was operated in negative electrospray ionisation (ESI-) mode. Flow rate was 0.25ml/min and an injection volume of 2μL was used. Gradient was as follows: 90% B; 70% B 11.5min; 60% B 12min; 100% 15min. The column was held at 90% B for 5mins between sample injections. MS data were acquired using a Dual Agilent Jet Stream ESI source operating at fragmentor and capillary voltages of 3500V and 125V, respectively. The nebulizer pressure was set at 40psi and the nitrogen drying gas flow rate was set at 10L/min. The drying gas temperature was maintained at 200°C. The Sheath gas temperature and flow were 300°C and 12L/min, respectively. The MS acquisition rate was 2 spectra/sec and m/z data range from 40-1000 were stored in profile mode. Dynamic mass axis calibration was achieved by continuous infusion of a reference mass solution using an Agilent 1260 isocratic pump (Agilent Technologies, G7110B) connected to the ESI source. All samples were run in a single LC-MS batch to limit variation from instrument effects.

### LC-MS data analysis

Analysis was performed using Agilent MassHunter ProFinder (version 10.0) in a similar manner to Cruickshank-Quinn et al. (24) Briefly, the “Batch Targeted Feature Extraction” and “Batch Recursive Feature Extraction’’ methods were used for the targeted and untargeted analyses, respectively. Formula targets were limited to Negative H- ion species and charge states limited to a range of 1-2. Mass and retention time (RT) tolerances were limited to ±10ppm+2mDa, and ±0.00%+0.5min, respectively. Features with an absolute height below 1,000 and saturation >20% were excluded. Detected metabolite features had to satisfy conditions in at least 33% of files in at least 1 sample group. Compounds detected in extraction blank samples and with %CV >20% in PooledQC samples were excluded from analysis. Metabolite abundances were calculated using areas integrated at the apex of selected chromatographic peaks. Values were exported as. CSV or. CEF files for further analysis and visualisation. Significantly altered entities were distinguished by paired t-test with a Benjamini-Hochberg FDR correction. Only metabolites with a fold change ≥1.5 and a p value <0.05 were considered significant.

### Metabolite annotation

Significantly altered metabolites were imported into Mass Profiler Professional (MPP, Version 15.0) ID Browser (version 10.0). Tentative metabolite IDs (25) were assigned using the Agilent MassHunter METLIN Metabolomics Database (curated in Agilent PCDL Manager, version B.08.00). Compounds were annotated in the first instance by Library/Database search, and by formula generation when there were no Library/Database hits. LC/MS tolerances for precursor ion m/z were ± 10ppm + 2mDa. Identification parameter (Database search, Molecular Formula Generation score) score weights were all evenly set to 40. Search results were limited to the 10 best hits per compound and scores above 70 were considered reliable. Features were annotated with tentative metabolite IDs assigned based on 1) score, 2) the presence of a commercialised standard, and 3) brief literature review to determine likelihood of metabolite’s impact on human monocyte function. Endogenous metabolites (as listed in HMDB) were preferred. Where no metabolite IDs or formulae were available, metabolite features were annotated by their mass and RT values (e.g., 601.2048@4.95).

#### Class prediction analysis

Partial Least Squares Discrimination Analysis (PLS-DA) and Random Forrest (RF) class predication analyses were carried out in Agilent Mass Profiler Professional (MPP). Both models were built using all 24 samples. The PLS-DA model used 2 components and was autoscaled. The RF model was built on 500 trees using 14 predictors and used a GINI variable importance calculation. Variable importance in projection (VIP) scores above 1 were considered significant.

### Flow cytometry

Dextran sediment (as a surrogate for whole blood) and isolated monocytes were analysed for purity, viability, and surface ANCA expression. 100μL of dextran sediment or 1x10^6^ purified monocytes were used for flow cytometry experiments. Red blood cells in dextran samples were lysed with 1mL of diluted BD lysis buffer before washing and staining. Cells were stained with CD14- PE-Vio770 (TÜK4), MPO-PE (2C7), and fixable viability dye eFluor450 for 30 mins in the dark. Cells were washed and immediately acquired on a FACSCanto II flow cytometer using BD FACSDiva™ software, and results analysed using Kaluza software (Version 2.1, Beckman Coulter, Inc.).

### Statistics

Statistical tests were carried out in GraphPad Prism software (Version 9.3.1). LPS-stimulated cells were compared to unstimulated using paired t-tests. Where multiple readouts were analysed, a Benjamini, Kreiger, and Yekutieli false discovery rate (FDR) correction (Q=1%) was performed. Pearson correlation was used to assess relationships between metabolites and flow/cytokine readouts. Results are only statistically significant (p <0.05) where specified.

## Results

### Optimised sample preparation of primary human monocytes for LC-MS analysis requires data normalisation to residual protein content

Given the dearth of standardised protocols for metabolomic analysis of primary cells, in particular primary blood monocytes, we first sought to develop an optimised sample preparation method. In a pilot study of 6 biological replicates, a combination of methanol-based extraction and ice bath sonication was the most effective extraction protocol in terms of numbers of detectable features and reproducibility (**Figure 1A**). This method provided excellent coverage of the monocyte metabolome. We found sample quenching and log transformation to be essential steps for sample preparation and data analysis, respectively (**Figure 1B**). Another essential [and challenging (26)] element of metabolomic analysis is data normalisation. After reviewing over forty biological and algorithmic data analysis strategies, the most efficient and consistent technique was an external scalar normalisation to residual protein content in the metabolite fraction. This was measured using a commercial BCA assay, with minor modifications. We first confirmed that methanol (MeOH) did not interfere with the assay system by comparing its performance to PBS-BSA solutions. MeOH correlated very well with PBS in both plate- and tube-based assays (**Figure 1C**, R^2^ = 0.9823, p<0.0001). Further, sample volumes as low as 10μL maintained consistency in protein measurements with this plate-based assay (**Figure 1D**). Normalising metabolite concentrations to BCA-corrected data improved the appearance of Principal Component Analysis (PCA) plots for the targeted analysis (**Figure 1E**). Residual protein content was also strongly correlated with cell number, more so than cell pellet and cellular DNA content (**Figure 1F**). Overall, the BCA protein assay was compatible with 80% MeOH and suitable for measurement of residual protein content measurements in metabolomic extractions. All subsequent results reported have been log_2_ transformed and BCA-normalised.

### Targeted metabolomic analysis reveals alterations in amino acids with ANCA stimulation

**Figure 2:**
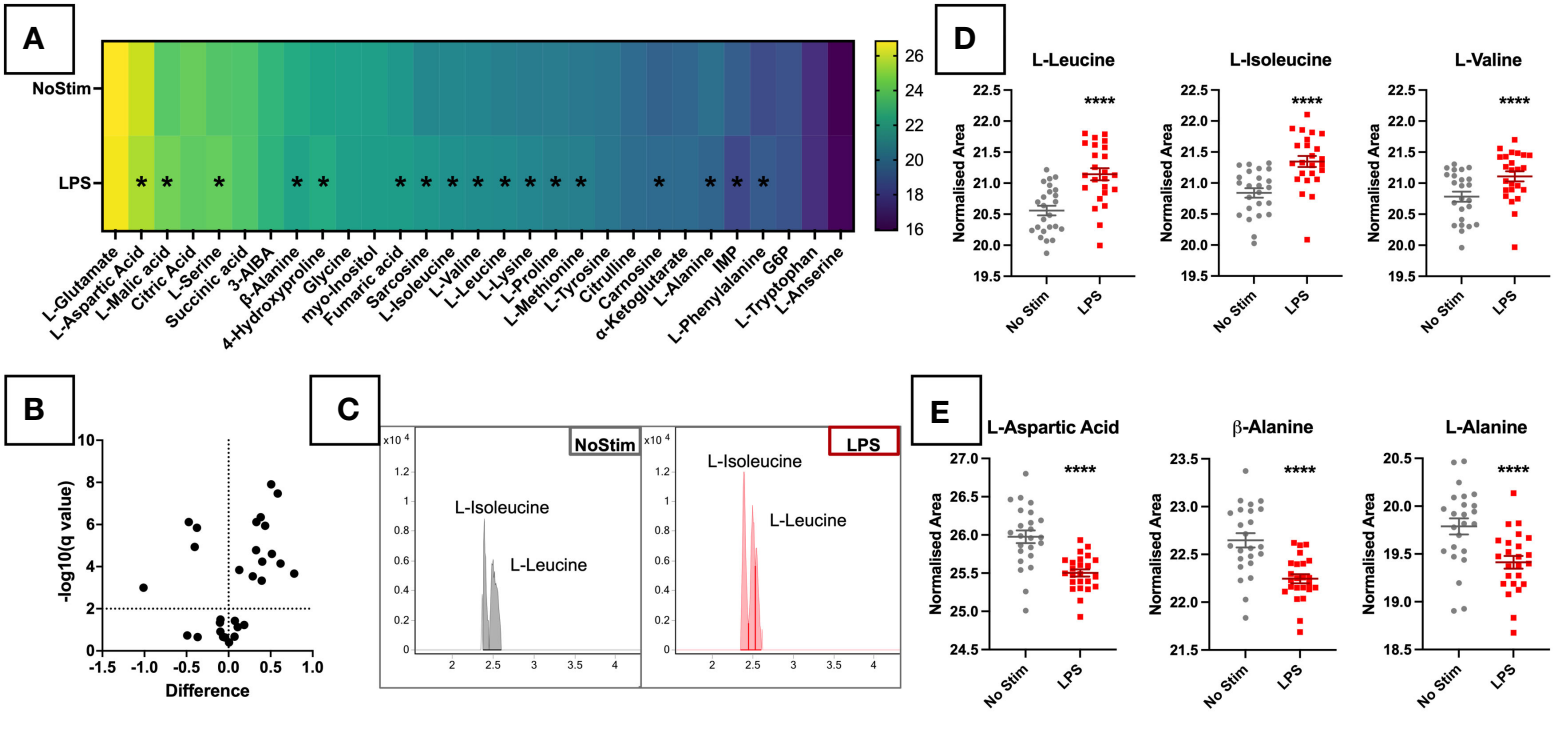
Targeted Metabolomic Analysis of LPS Stimulated Primary Monocytes. **(A)** CD14+ monocytes were stimulated for four hours with 200ng/ml LPS. Monocytes were analysed by LC-MS and targeted metabolomic analysis of 53 metabolites was completed in ESI-. **(A)** Heatmap of targeted metabolites in LPS-stimulated monocytes. LPS-stimulated monocytes were analysed by LC-MS and targeted metabolomic analysis of 53 metabolites was completed. 29 of the 53 metabolites were detected in experiment samples. Intergroup values are reported as BCA and Log2 normalised AUCs. Significant metabolites of varying degrees are marked with *. **(B)** Volcano plot analysis of targeted metabolites in LPS-stimulated monocytes. Metabolite features were analysed by Mann-Whitney paired test with a Benjamini-Hochberg FDR correction, and features with fold change ≥1.5 and p value <0.05 were considered significant. Fold change and p-value thresholds are shown in dotted lines on the X and Y axes, respectively. EICs for leucine and isoleucine are displayed in **(C)**, and sample plots of BCAAs **(D)** and metabolites reduced in the LPS-stimulated cells **(E)** are shown in individual dot plots. TIC, Total Ion Chromatograms; ESI, Electrospray Ionisation; BCAAs, Branched-Chain Amino Acids; EIC, Extracted Ion Chromatogram.*** p < 0.001.

Twenty-nine of the 53 synthetic standard metabolites were detected in experimental samples, and forty in PQCs. In the targeted analysis changes between treatment groups were relatively small at this early time point, however, 17 metabolites out of the 29 did reach statistical significance. Thirteen were upregulated in LPS-treated cells, and four downregulated (**Figure 2A**). The heatmap of AUC values (**Figure 2B**) demonstrates changes in a wide range of metabolites upon LPS stimulation. These mostly comprised amino acids, as well as TCA cycle metabolites (fumaric acid, L-malic acid) and nucleotides (inosine 5’-monophosphate [IMP]). Among these, the branch-chained amino acids (BCAAs) leucine, isoleucine (**Figure 2C**) and valine were among the most significantly increased by LPS stimulation (**Figure 2D**). In contrast, aspartic acid and two alanine isomers were depleted upon LPS activation (**Figure 2E**). These results demonstrate that, metabolic changes can be detected as early as four hours post LPS stimulation.

### Inositol and glucose metabolism are altered in monocytes stimulated with LPS

**Figure 3:**
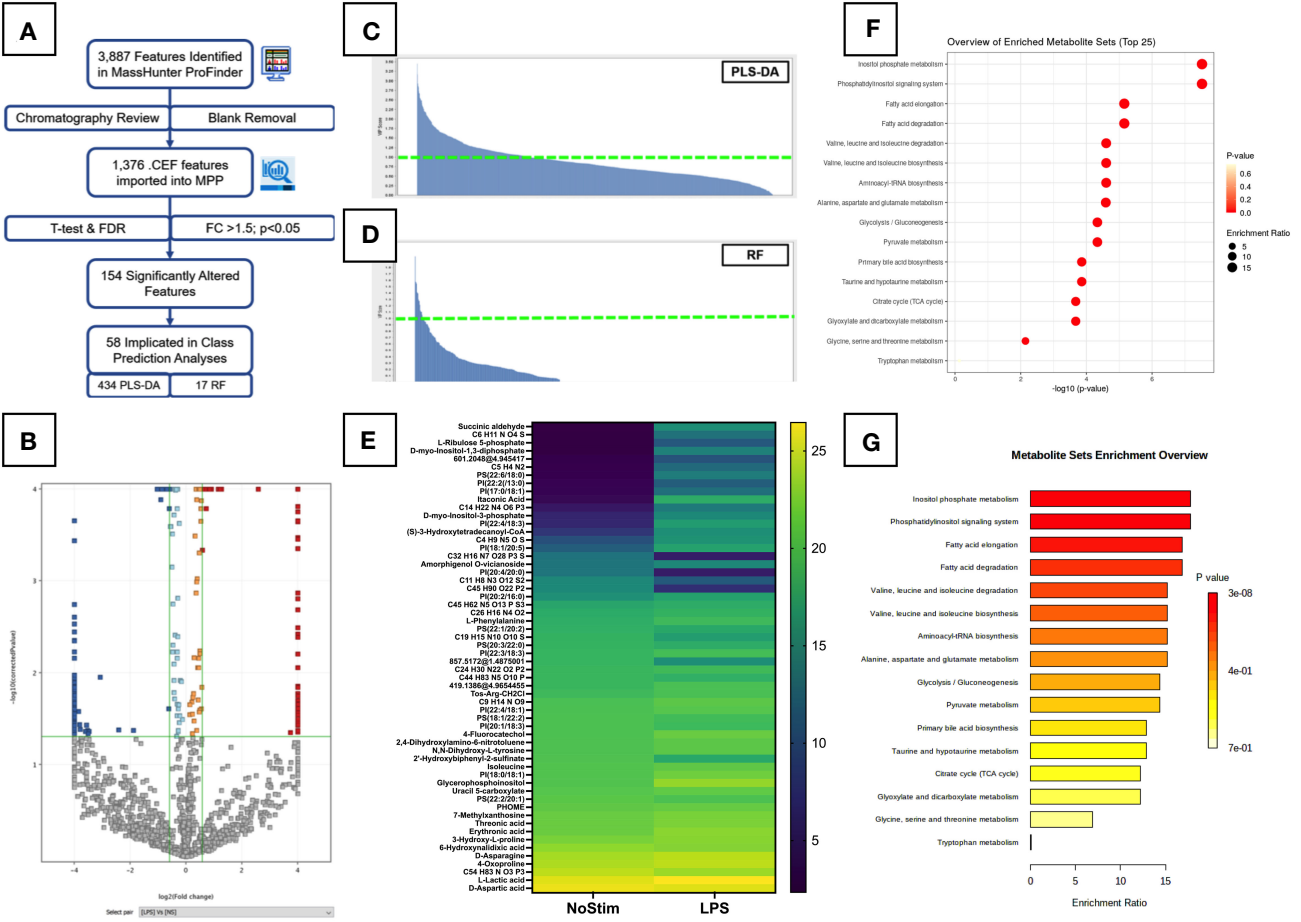
Untargeted Metabolomic Analysis of LPS Stimulated Primary Monocytes. **(A)** Summary workflow for untargeted metabolomic analysis with numbers of features/entities specified at each stage. **(B)** Volcano plot of LPS-treated monocytes are shown with significantly upregulated features highlighted in red and downregulated features in dark blue. Light blue and orange features did not meet the fold change threshold limits, and grey features were insignificant. Fold change and p-value thresholds are shown in green lines on the X and Y axes respectively. Class prediction analyses of significantly altered untargeted metabolites carried out by PLS-DA **(C)** and Random Forrest analysis **(D)**. Entities with a VIP score >1 are plotted on the heatmap in **(E)**. Pathway analysis of significantly altered metabolites identified in untargeted analysis was performed on the online MetaboAnalyst platform (Version 5.0, https://www.metaboanalyst.ca/). Results are displayed as dot plots **(F)** and bar chart **(G)**.

For a more global overview of the metabolic changes, we performed untargeted analysis of LPS-stimulated monocytes. A summary of the untargeted workflow is shown in **Figure 3A**. A total of 1,376 features were detected after manual chromatography review and removal of peaks present in extraction blank samples. These were assigned tentative metabolite IDs. Out of 1,376 features detected, 154 were significantly altered in the LPS-treated monocytes (**Figure 3B**): 87 with decreased abundance, and 67 with increased levels compared to unstimulated cells. Of note, itaconic acid, an important modulator of inflammatory responses in macrophages (27), was found to be highly upregulated in LPS-activated cells at this early time point.

To better understand the specific influence and fundamental relations of these features, class prediction analyses were carried out using PLS-DA and RF analysis. Both models had an overall accuracy of 97.92%: 95.83% for unstimulated cells, 100% for the LPS-treated group. VIP scores and heatmaps for PLS-DA and RF are shown in **Figures 3C**, **D**, respectively. The PLS-DA model returned 434 features with a score above one, and 47 above two. The RF model had 17 features >1, with six of these common to both analyses. Four of these features were assigned as: L-lactic acid, uracil 5-carboxylate, D-myo-Inositol-1,3-diphosphate, and PI(38:4). A heatmap of the 58 influential metabolites is displayed in **Figure 3E**. We see changes in a number of lipid-like molecules, particularly several glycerophosphoinositols (PI) and glycerophosphoserine (PS) species, as well as a number of amino acids.

Pathway analysis for all 1,367 assigned features detected in the untargeted analysis was carried out using MetaboAnalyst (Version 5.0, https://www.metaboanalyst.ca/). **Figure 3F** shows annotated bubble plots for the most significantly altered metabolites in LPS-stimulated monocytes. Phosphatidylinositol signalling and inositol phosphate metabolism were the most significantly enriched pathways in LPS-treated cells, however, both of these pathways had a very low pathway coverage and thus a low pathway impact score. Other pathways previously reported to be altered by LPS were also seen at this early time point, including the Pentose phosphate pathway (PPP), glycolysis/gluconeogenesis, and nicotinate and nicotinamide metabolism. This suggests increased utilisation of glucose by early branches of the glycolysis pathway. Quantitative enrichment analysis confirmed the impact of LPS on the inositol phosphate metabolism and phosphatidylinositol signalling pathways (**Figure 3G**). BCAA degradation and biosynthesis were also impacted, confirming the findings of the targeted analysis (**Figure 2D**). These results must be considered cautiously, as metabolite annotations were only available for a small subset of the metabolites, and at a lower confidence level (28) than those in the targeted analysis. Additional analyses are needed to improve coverage of these metabolite pathways and confirm their importance LPS-activated inflammation.

### Targeted metabolomics and links to surface MPO and cytokine production

**Figure 4:**
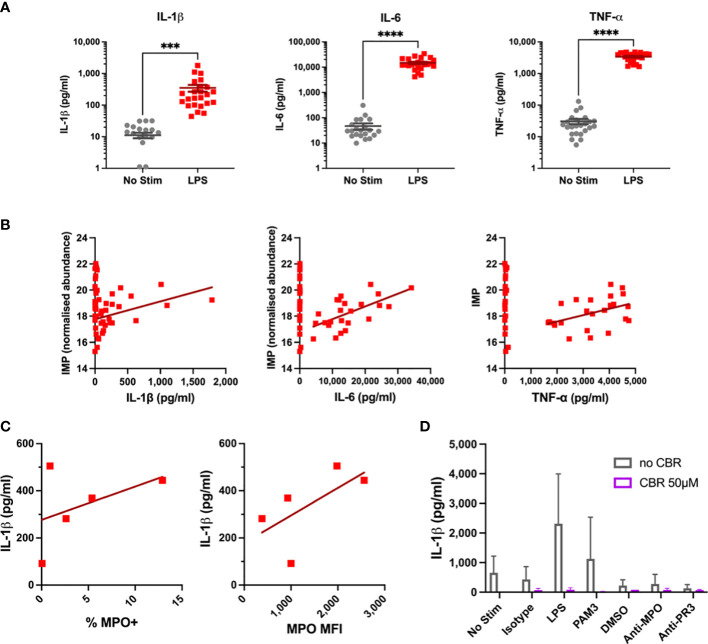
Cytokine and Flow Results for LPS-Stimulated Monocytes. **(A)** IL-1β, IL-6, TNF-α levels were measured in supernatants of monocytes by ELISA after four hour LPS stimulation. **(B)** Cytokine levels are correlated with IMP concentration in primary monocytes. **(C)** Correlation of baseline MPO surface expression with IL-1β production in stimulated primary monocytes. **(D)** Effects of serine synthesis pathway inhibition on IL-1β production in LPS-Stimulated Monocytes. *** p < 0.001, **** p < 0.0001.

We next wanted to determine if these alterations in monocyte metabolism in response to LPS occur in line with cellular activation. Cytokine production is a key function of monocytes, and significant increases in IL-1β, IL-6, and TNF-α were observed after four hours of LPS stimulation (**Figure 4A**). Increased cytokine production correlated with levels of a number of metabolites. Of note, inosine 5’-monophosphate (IMP) significantly correlated with all three cytokines (**Figure 4B**). Further correlations were found for a number of amino acids, including the BCAAs, L-lysine, L-methionine, and L-phenylalanine. Despite this, these correlations had rather low R^2^ values, likely owing to the short timescale of these experiments. Surface expression of myeloperoxidase (MPO) was measured in a subset of samples prior to stimulation. The percentage of MPO-positive monocytes and median fluorescence intensity (MFI) of MPO appeared to correlate with subsequent IL-1β production upon LPS stimulation (**Figure 4C**), suggesting that surface MPO levels may act as a surrogate marker for cellular activation. Serine has previously been shown to be essential for IL-1β production in LPS-stimulated murine macrophages (29). Given the increase in glycolysis upon LPS activation, it is feasible that flux through the serine pathway could be increased to support IL-1β production in human monocytes. Inhibiting serine production with CBR-5884 for 30 mins prior to stimulation completely abrogated IL-1β production (**Figure 4D**). These data suggest that CBR-5884 can inhibit IL-1β production by limiting serine synthesis. These data present clues to the emergent changes to monocyte metabolism in response to LPS.

## Discussion

To date, most investigations of monocyte metabolomics have been carried out at later time points, such as >24h stimulations, a steady state in terms of metabolism. Here we defined the inchoate monocyte response to LPS-stimulation after four hours and provide detailed protocols for effective reproducibility of these works. Our method limits hands-on sample time with a quick single phase methanolic extraction and a single complementary and comprehensive LC-MS method.

First, we describe optimised sample preparation protocols for monocyte metabolomic investigations. A methanol-based extraction and gentle cell lysis method was found to be optimal (**Figure 1A**). This method has been effective in adherent cells (30, 31), and is commonly used in cellular metabolomics investigations (32). Harvesting cells by plate scraping or trypsin-EDTA can influence the intracellular metabolome (33). Suspension cells such as monocytes can be easily extracted by adding cold extraction solvent directly to stimulation plate(s) before being transferred to the tube. This allows thorough extraction of any metabolites from remaining cells, limiting contamination from cell supernatants (exometabolome).

Experimental design and technical variation can affect the outcomes of metabolomics studies. As such, a number of data normalisation methods have been developed to correct for these errors (26, 34). We found that residual protein in the metabolite fraction measured by BCA protein assay was the optimum normalisation method. This method has been successfully used in previous work (35) and, in contrast to prior reports (26), protein levels did correlate with metabolite abundances in this analysis. Extending the (BCA assay) sample incubation time to 18 hours improved detection of small protein volumes, and the plate-based assay facilitates minimal sample loss, preserving low volume samples for LC-MS analysis (**Figure 1D**). We also found that acetonitrile was compatible with the commercial BCA assay (data not shown). The compatibility of other metabolite extraction solvents should be explored further.

Previously published LC-MS methods for monocyte metabolomic profiling were carried out by commercial metabolic profiling companies (15, 22) which combine RP and HILIC liquid chromatography to measure four metabolite subfractions. While we initially sought to replicate these techniques, the HILIC ESI- method consistently returned the greatest numbers of viable metabolite features. HILIC methods can detect a larger number of molecular features than RPLC and at higher abundances (36). Certain lipid classes in myeloid cells have been reliably detected by HILIC single phase extraction (31), and a recent publication noted the detection of over 1,900 lipid species across 26 subclasses from a single 12-minute HILIC LC-MS method (37). These are largely polar, small lipids, and the relevance of these compounds to monocyte inflammatory responses should be investigated in future profiling experiments.

Using these optimised protocols, we found novel metabolomic changes in primary monocytes after four hours of LPS stimulation. There have already been a number of investigations of monocyte metabolism at 24 hours (15, 16, 18, 21, 22), a timepoint which represents steady state monocyte metabolism. Zhu et al. (21) investigated a sepsis model in monocyte-like THP1 cells through activation, deactivation, and resolution of LPS-induced inflammation. At eight hours cells were already sufficiently activated, with significant changes across a range of metabolic pathways. Given the broad range of metabolic and inflammatory responses to LPS, longer timepoints pose a threat of artefacts from autocrine effects. TNF-α signalling can alter a broad range of metabolic pathways and affect insulin sensitivity in obesity (38). Conversely, succinate can induce IL-1β expression to promote inflammation (1). To preserve the LPS-specific effects of metabolism and the additional reasons above, we opted to investigate the early changes in monocyte metabolism after four hours of stimulation.

A number of amino acids were enriched in these cells, including the BCAA isomers leucine and isoleucine. Leucine in particular, is key for inflammatory cytokine production in these cells. Leucine influx *via* SLC7A5 contributes to metabolic reprogramming and IL-1β production in primary monocytes (39). This receptor is also upregulated on monocytes of RA patients where it correlated with disease severity markers (39). Decreased intracellular L-aspartate upon LPS activation has been reported at 6 hours (12), and is exacerbated by glucose deprivation. However, Arts et al. (18) found that aspartic acid was depleted in medium from trained monocytes. This may indicate increased breakdown of aspartate to produce more bioactive amino acids such as those described above. The precise reasons for depletion of aspartate and alanine metabolites should be further explored at different time points.

Serine is another amino acid important for inflammatory cytokine production. In murine macrophages serine production was found to be essential for IL-1β (and TNF-α) production upon LPS activation, and inhibiting serine synthesis abrogated inflammatory cytokine release (29). CBR-5884 inhibits 3-phosphoglycerate dehydrogenase (PHGDH) – the first step of the *de novo* serine synthesis pathway which directly branches off from glycolysis (40). The increased glycolysis reported in LPS-stimulated monocytes could therefore be fuelling serine production, and subsequent IL-1β release. In macrophages the one-carbon pool facilitates epigenetic changes during proinflammatory macrophage (M1) differentiation, fed in part by serine synthesis (41, 42). However, serine was not increased in LPS-stimulated monocytes in this work, and has not been reported in more long-term stimulations. We did find that the inhibitory effects of CBR-5884 on IL-1β production were lost when cells were stimulated for 18 hours (data not shown). Whether serine synthesis has a prolonged role in cytokine production in monocytes should be a focus of future research.

The untargeted analysis revealed further novel metabolic pathways perturbed by LPS in monocytes. Inositol phosphate metabolism and phosphatidylinositol signalling were the top two metabolic pathways implicated by LPS activation (**Figures 3F, G**). Myo-inositol was increased at four hours (**Figure 2A**), and was also found to be increased after 24 hours of LPS stimulation (15, 22). Other pathway metabolites were also increased (**Figures 3E**), indicating that these pathways are important for early monocyte activation. Additional validation experiments are required to confirm the influence of these metabolic pathways on monocyte function upon LPS activation.

Fatty acid metabolism was also significantly altered at this early time point. Other fatty acids have been implicated at later time points (12, 15, 22), and Fatty Acid Oxidation (FAO) has been shown to be essential for CCL20 production by RA monocytes (11). In low glucose/oxygen conditions (or where competition for glucose/oxygen is high) monocytes increase FAO *via* oxidative phosphorylation to meet their energy demands (12). This broad increase may indicate a metabolic emergency, whereby multiple energy generating pathways become activated prior to a specific metabolic (and associated inflammatory) response. Recently lipid metabolism was shown to be important in COVID-19 (43), and these pathways should be investigated further.

Pro-resolving M2 macrophages preferentially utilise FAO (as well as OXPHOS and glutaminolysis) as a means of energy generation (44, 45), though these cells are unlikely to be undergoing pro-resolving differentiation at this early time point. Itaconic acid is a well-known anti-inflammatory metabolite increased in activated macrophages (27). Increased itaconic acid has also been reported in LPS-stimulated monocytes (15, 22), and was found to be increased at our early time point (**Figure 3E**). Understanding the role of this metabolite prior to macrophage differentiation should be a focus of future work.

We report a novel increase in surface MPO expression which appears to link to inflammatory activation of primary monocytes (**Figure 4C**). Little is known about surface MPO expression and its relationship to metabolism. Our group has previously shown differences in MPO expression between monocyte subsets, and subsequent differences in pro-inflammatory cytokine release (46). Despite this, we found only a mild relationship between inflammatory cytokine production and metabolism. Additional activation markers should be explored to confirm the link between metabolic activation and upregulation of inflammatory responses.

## Data availability statement

The data presented in the study are deposited in the MetaboLights repository (https://www.ebi.ac.uk/metabolights/index), accession number MTBLS6730.

## Ethics statement

The studies involving human participants were reviewed and approved by The St. James’ Hospital (SJH)/Tallaght University Hospital (TUH) Joint Research Ethics Committee (REC). Written informed consent for participation was not required for this study in accordance with the national legislation and the institutional requirements.

## Author contributions

EL designed and performed experiments, analysed data, and wrote the manuscript. IB performed experiments and assisted in data analysis. LS performed experiments. MMcE assisted in data analysis. GB, MAL, and HK supervised the project and reviewed the manuscript. HK performed experiments and assisted in data analysis. All authors contributed to the article and approved the submitted version.
